# Association of serum CTRP4 levels with vascular endothelial function in patients with type 2 diabetes mellitus: CTRP4 ameliorating inflammation, proliferation and migration in human umbilical vein endothelial cells

**DOI:** 10.1007/s00592-023-02228-3

**Published:** 2024-01-29

**Authors:** Jie Gao, Mai Re YanMu Rouzi, Huihui Zhang, Xinghua Cai, Bilin Xu, Jun Lu, Tao Lei

**Affiliations:** 1 Department of Endocrinology, Putuo Hospital, Shanghai University of Traditional Chinese Medicine, 164 LanXi Road, Shanghai, 200062 China; 2 School of Medical and Life Sciences, Chengdu University of Traditional Chinese Medicine, Chengdu, China; 3 Shanghai Putuo Center School of Clinical Medicine, Anhui Medical University, Hefei, Anhui China

**Keywords:** Endothelial function, CTRP4, Flow-mediated dilation, Type 2 diabetes mellitus

## Abstract

**Objective:**

We investigated the correlation between serum C1q/TNF-related protein 4 (CTRP4) level and flow-mediated dilation (FMD) in patients with type 2 diabetes mellitus (T2DM), and evaluated the biological effects of CTRP4 on human umbilical vein endothelial cells (HUVECs).

**Methods:**

A group of 165 patients diagnosed with T2DM were included in this study. Endothelial function was measured with the examination of brachial artery FMD. ELISA kit was used to measure the levels of CTRP4 in serum. HUVECs were stimulated with recombinant CTRP4 protein to assess its biological functions.

**Results:**

The levels of CTRP4 showed a significant variation among three groups based on FMD tertiles (*p* = 0.001). What’s more, FMD had a significant difference among three CTRP4 tertile groups (*p* < 0.05) and was negatively related to serum CTRP4 levels (*r* = −0.270, *p* < 0.001). In T2DM patients, logistic regression analysis demonstrated that CTRP4 was the primary influence factor of low FMD (*p* < 0.01). In receiver operating characteristic curve analysis, the area under the curve of CTRP4 for predicting low FMD was 0.66 (95%CI 0.58–0.75). When stimulated HUVECs with recombinant CTRP4 protein, we found that CTRP4 could concentration-dependently ameliorate proliferation and migration of HUVECs in wounding healing and transwell assay. This protein could also decrease the expression of IL-6 and TNF-α and promote the release of NO in HUVEC supernatants, with suppression of NF-κB and STAT3 phosphorylation.

**Conclusions:**

Serum CTRP4 concentrations were negatively associated with FMD. CTRP4 alleviated proliferation, migration and inflammation in HUVECs through the suppression of NF-κB and STAT3 signaling pathways.

## Introduction

The development of atherosclerosis often begins with endothelial dysfunction, which is frequently found in type 2 diabetes mellitus (T2DM) [1, 2]. Vascular endothelial impairment can be caused by hyperglycemia through various mechanisms, such as oxidative stress, chronic inflammation, the generation of nonenzymatic advanced glycation end products (AGEs) and apoptosis [3, 4]. Recently, measurement of flow-mediated dilatation (FMD) has been widely recognized as a useful, low-risk and non-intrusive technique for the evaluation of endothelial function [5–7]. It reflects the response to the endothelium-derived nitric oxide (NO) caused by reactive hyperemia after the recovery from acute occlusion in the upper limb and has been believed to be an independent influencing factor of cardiovascular disease [8–10].

C1q/TNF-related proteins (CTRPs) superfamily (CTRP1-CTRP15), first found in 2004 by Harvey Lodish and his colleagues [11], has been identified as being sequenceally and structurally related to adiponectin [12]. Studies have demonstrated that CTRPs perform multiple functions in regulating glucose/lipid metabolism and immune-inflammation [13–16]. In addition, a growing body of evidence has proposed that CTRPs were associated with the progression and prognosis of coronary artery disease (CAD) [17–22]. C1q/TNF-related protein 4 (CTRP4), the unique member of CTRPs family with two globular C1q domains connected by a short linker [23], was mainly distributed in brain, adipose tissue, and also existed in circulation [24]. CTRP4 attracted wide attention due to its diverse effects, including reducing appetite, regulating inflammatory response, modulating glucose and lipid metabolism [23, 25–28]. Our previous study reported that T2DM subjects with CAD had higher serum CTRP4 levels than subjects without CAD, suggesting the reference value of CTRP4 for the occurrence of CAD in patients with T2DM [29]. However, there is still a lack of understanding of how CTRP4 is related to endothelial function, especially in diabetic environment in which vascular endothelium is likely impaired.

Hence, this study was carried out to reveal the association between serum CTRP4 concentrations and vascular endothelial function in individuals diagnosed with T2DM. To further evaluate CTRP4 effects on endothelial cells in vitro, we stimulated HUVECs using recombinant CTRP4 protein and examined proliferation, migration and inflammation behaviors of HUVECs, with confirmation of related pathways.

## Materials and methods

### Study design and patient enrollment

**Fig. 1:**
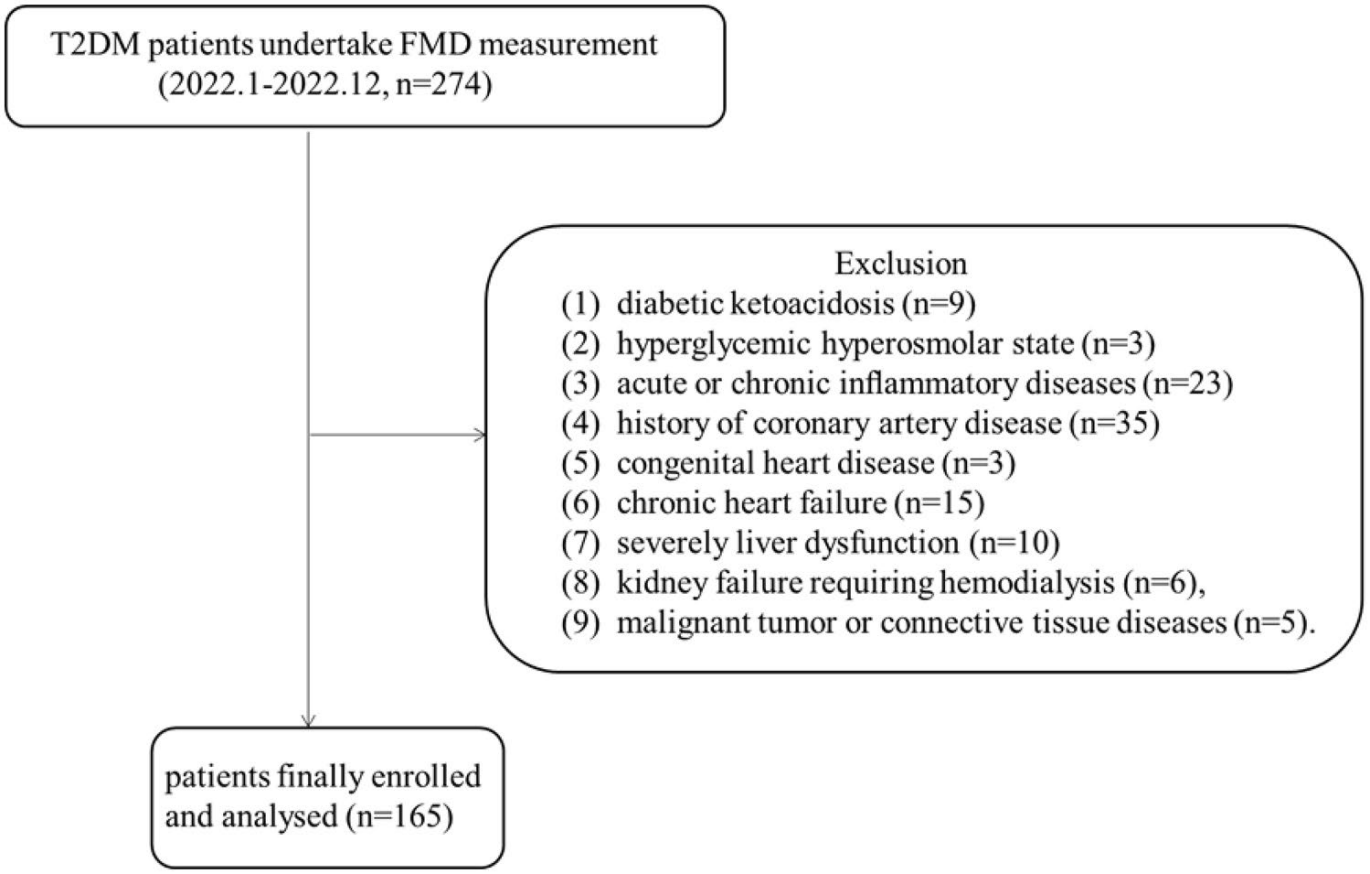
Flowchart of the project process

This study was conducted in a cross-sectional manner. We initially recruited a sum of 274 individuals diagnosed with T2DM and taken FMD measurement in Putuo hospital from January 2022 to December 2022. In order to achieve the aim of research, we excluded patients with following conditions: (1) diabetic ketoacidosis, (2) hyperglycemic hyperosmolar state, (3) acute or chronic inflammatory diseases, (4) history of coronary artery disease, (5) congenital heart disease, (6) chronic heart failure, (7) severely liver dysfunction (aspartate aminotransferase or alanine transaminase ≥ 3 times than upper limit of normal), (8) kidney failure requiring hemodialysis and (9) malignant tumor or connective tissue diseases. Ultimately, a total of 165 participants were recruited for this investigation **(**Fig. 1**)**.

After obtaining written consent from all participants, the study was conducted at Putuo hospital, which is affiliated to Shanghai University of Traditional Chinese Medicine.

### Anthropometric and clinical laboratory measurements

A medical inquiry included disease history and drug treatment of all patients was recorded upon admission. Then, we performed a physical examination concerning the measurement of blood pressure (BP), height, weight and other anthropometric parameters. BP was measured twice when patients were supine, and then averaged. Body mass index (BMI) was calculated as dividing the weight (kg) by the square of the height (m^2^). Blood samples were taken in the morning after fasting overnight, and biochemical indexes such as blood glucose, lipid profiles, C reactive protein (CRP) and creatinine were detected by automatic biochemical analyzer (Beckman Coulter AU5800). High-performance liquid chromatography was employed to measure HbA1c by using the Tosoh Automated Glycohemoglobin Analyzer HLC-723G11. Serum levels of CTRP4 were evaluated using a specific ELISA kit (Raybiotech, USA) according to the protocols provided by the manufacturer. CTRP4 levels were presented as ng/ml.

Patients with systolic blood pressure (SBP) exceed 140 mmHg, diastolic blood pressure (DBP) surpass 90 mmHg [30], consuming antihypertensive drugs, or having a record of hypertension were defined as hypertension. Type 2 diabetes mellitus was defined according to the American Diabetes Association in 2021 [31], which included the fasting blood glucose ≥ 7.0 mM, 2 h postprandial blood glucose ≥ 11.1 mM and/or HbA_1c_ ≥ 6.5% or the administration of blood glucose-lowering medication.

### Brachial flow-mediated dilation measurement

After lying in a supine position for more than 10 min at room temperature, the assessment of flow-mediated dilation (FMD) was conducted on brachial artery in the upper right arm. This evaluation was performed using a high-resolution ultrasound device equipped with a 10 MHz linear array probe and the UNEX EF18VG (Nagoya, Japan) Imaging System, adhering to the recommended protocol. In brief, baseline diameter of brachial artery was determined, followed by the inflation of a blood pressure cuff on patient's right upper arm to a pressure of 200 mmHg and then deflated after 5 min. The maximum diameter at the same region was recorded after deflation. FMD was determined by the equation: [(maximum diameter—baseline diameter)/baseline diameter] × 100%. An experienced ultrasound doctor, who was blinded to the study design and clinical parameters of the participants, carried out the entire procedure.

### Cell culture and experimental protocol

Human umbilical vein endothelial cells (HUVECs) were purchased from Zhongqiao Xinzhou Biotechnology Co (Shanghai, China) and cultured in an atmosphere containing 5% CO_2_ at 37 ℃ in Endothelial Cell Medium (ECM), supplemented with 5% fetal bovine serum (FBS) and 1% penicillin–streptomycin. HUVECs at the density of 5 × 10^5^ cells/well were cultured in 6-well plates. Cells stimulated with 10, 100, 500 ng/ml recombinant human CTRP4 protein (8357-TN-050, RD, USA) were set as the CTRP4 groups.

### Wound healing assay

HUVECs were grown to 90% confluence in 6-well plates and starved for 12 h. We made a scratch with a 200 μl pipette tip and incubated cells in fresh FBS-free ECM, with the treatment of CTRP4. The wound width of each dish was observed under a microscope (Olympus Corporation) in three randomly fields at 0 h and 12 h. Wounding healing rate (%) was calculated by the formula: (initial wounding area-12 h wounding area)/initial wounding area × 100%.

### Cell migration assay

Cell motility was detected using a modified assay with a pore size of 8 μm in 24-well transwell plates (Millipore, MA, USA). Briefly, 200 μL cell suspension were seeded in the upper chamber and 750μL ECM containing CTRP4 recombinant protein were added into the lower chamber. The chamber was incubated for 24 h at 37 ℃ in 5% CO_2_ incubator; then, the migrated cells were stained with crystal violet and analyzed. The cells were counted using a microscope (Olympus Corporation, Japan) in five distinct fields in three independent experiments.

### Enzyme-linked immunosorbent assay (ELISA)

The levels of interleukin-6 (IL-6), tumor necrosis factor-*α* (TNF-*α*) and nitric oxide (NO) in HUVEC supernatants were detected by the human IL-6 ELISA Kit (EK0410, Boster, Wuhan), TNF-α ELISA Kit (EK0525, Boster, Wuhan) and NO ELISA Kit (A013-2, jiancheng biotechnology, Nanjing), according to the manufacturer’s instructions.

### Western blot analysis

Total proteins from HUVECs were extracted using RIPA lysis buffer (Thermo Fisher Scientific, USA) containing 1% PMSF (5872 s, cell signaling technology, USA). The concentrations of proteins were measured using a BCA Kit (Beyotime Biotechnology, China). Equal amounts of protein extracts were separated on 7.5% SDS-PAGE (IPFL00010, Merck, Germany) and transferred to a polyvinylidene difluoride membrane (Millipore Sigma, USA). Anti-STAT3 (9139 s, cell signaling technology, USA), anti-phosphorylated-STAT3 (9145 s, cell signaling technology, USA), anti-p65(8242 T, cell signaling technology, USA), anti-phosphorylated-p65 (3033 s, cell signaling technology, USA) and *β*-actin (3700 s, cell signaling technology, USA) were incubated to identify specific proteins, which were then visualized with an electrochemiluminescence (ECL) reagent (LSW3515, LAISI Biotechnology, Shanghai).

### Statistical analyses

By using Kolmogorov–Smirnov test, continuous data were assessed for the normality. Continuous values that follow a normal distribution were represented by the mean ± standard deviation (SD). In cases where the values did not obey a normal distribution, the variables were displayed as the median (interquartile range). Frequency (percentage) was used to summarize categorical data. Differences among three groups were evaluated by One-way ANOVA or Kruskal–Wallis test as appropriate. *χ*^2^ test or Fisher’s exact test were employed for categorical data. In accordance with the levels of FMD, the subjects were categorized into three groups: Low FMD group ≤ 3.7%; Middle FMD group, 3.7–5.6%; High FMD group ≥ 5.6%. Logistic regression was performed to evaluate the connection between CTRP4 and low FMD. To examine CTRP4 for sensitivity and specificity in predicting low FMD in T2DM, a receiver operating characteristic (ROC) curve analysis was carried out. The optimal cut-off was calculated according to maximal Youden index. The areas under the curves (AUCs) for different Models and CTRP4 were compared by the MedCalc statistical software. A criterion was established with a significance level of *p* < 0.05 for both tails. All data analyses were performed with IBM SPSS Version 22.0 software (IBM SPSS Inc, Chicago, IL, USA).

## Results

### Baseline characteristics of all the patients

**Fig. 2:**
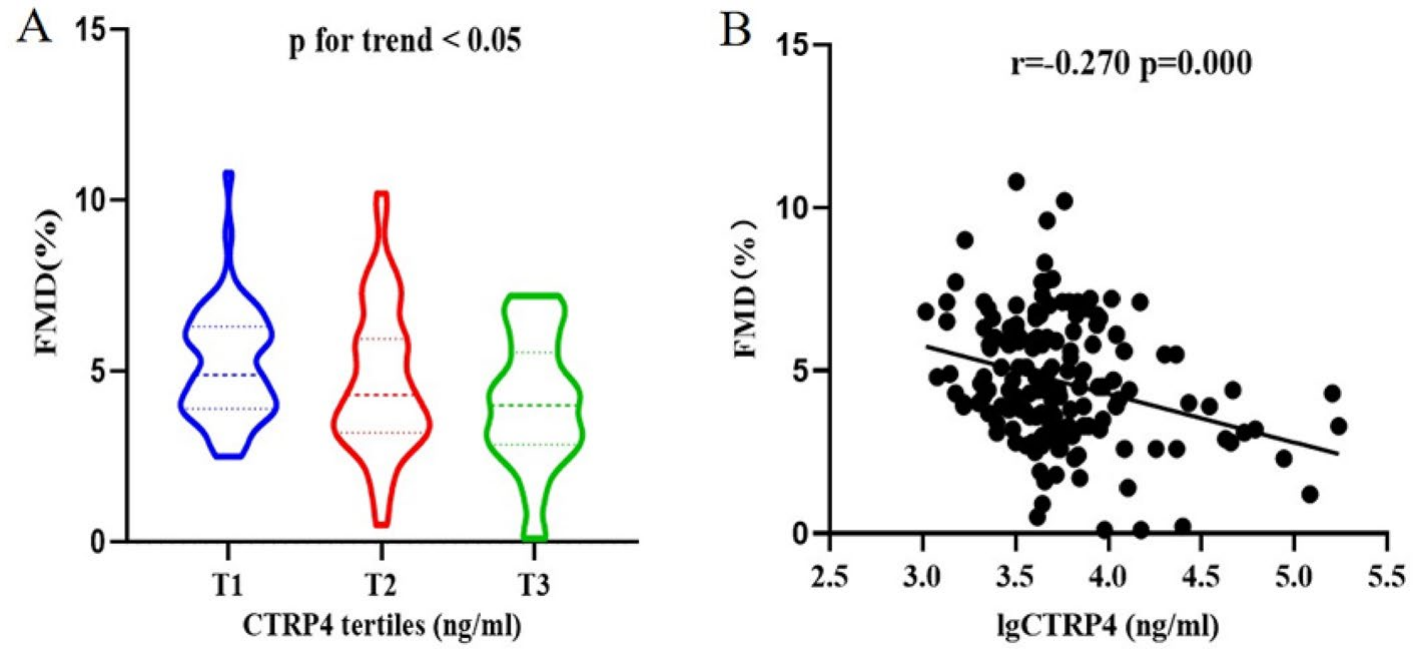
Association between CTRP4 and FMD. **A** FMD distribution in CTRP4 tertiles in all patients; **B** Correlation between FMD and CTRP4

Baseline characteristics and clinical indexes of the study population among three FMD groups are shown in Table 1. As compared to low FMD group, patients in middle and high FMD group had lower SBP (*p* for trend < 0.05). Despite the proportion of participants using calcium channel blocker or diuretic was higher in the low FMD group, the percentage of patients taking antihypertensive drugs failed to differ significantly among three groups (*p* = 0.059). Remarkably, significant differences regarding serum CTRP4 levels were observed across the three FMD groups (*p* for trend < 0.01). When categorizing all these participants into three groups according to CTRP4 tertiles as follows: tertile 1, ≤ 4.06 ng/ml; tertile 2, 4.06–6.24 ng/ml; tertile 3, ≥ 6.24 ng/ml, median FMD across the three groups were 4.9 (3.9–6.3)%, 4.3 (3.2–6.0)% and 4.0 (2.9–5.6)% respectively, indicating a significant difference (*p* = 0.016) **(**Fig. 2A**)**. Moreover, FMD was inversely correlated to serum CTRP4 levels in all the diabetic patients (*r* = −0.270, *p* < 0.001) **(**Fig. 2B**)**.

**Table 1:** Characteristics of diabetic patients sorted by FMD

	Low FMD (*n* = 56) ≤ 3.7%	Middle FMD (*n* = 54) 3.7–5.6%	High FMD (*n* = 55) ≥ 5.6%	*p* for trend
Male (*n*, %)	41 (73.2%)	33 (61.1%)	38 (69.1%)	0.386
Age (years)	64 (60–71)	64 (60–68)	62 (51–67)	0.091
BMI (kg/m^2^)	24.26 ± 3.33	24.99 ± 3.89	23.97 ± 3.29	0.301
Smoking (*n*, %)	16 (28.6%)	12 (22.2%)	19 (34.5%)	0.362
Drinking (*n*, %)	10 (17.9%)	5 (9.3%)	12 (21.8%)	0.175
DM duration (months)	120 (8–240)	120 (0–180)	72 (0–201)	0.903
Hypertension (*n* %)	40 (71.4%)	32 (59.3%)	27 (49.1%)	0.055
SBP (mmHg)	140 (129–153)	136 (125–148)	130 (125–138)	0.047
DBP (mmHg)	81 (80–90)	86 (80–90)	80 (77–90)	0.170
FBG (mmol/l)	7.19 (5.65–10.40)	7.95 (6.93–10.85)	8.75 (5.91–10.85)	0.709
HbA1c (%)	9.70 (7.90–11.85)	9.95 (8.40–11.83)	9.95 (8.23–12.05)	0.966
PBG (mmol/l)	15.32 ± 5.44	15.77 ± 7.92	15.15 ± 6.31	0.887
TC (mmol/l)	5.04 (3.99–6.06)	4.66 (3.67–5.64)	4.70 (3.89–5.85)	0.369
TG (mmol/l)	1.60 (0.98–2.86)	1.59 (0.99–2.12)	1.37 (1.01–2.05)	0.444
HDL-C (mmol/l)	1.10 (0.94–1.26)	1.04 (0.85–1.34)	1.00 (0.88–1.24)	0.492
LDL-C (mmol/l)	3.12 (2.33–3.95)	2.95 (2.35–3.56)	3.27 (2.44–4.02)	0.730
Serum creatinine (umol/l)	66 (50–79)	62 (52–83)	56 (47–72)	0.811
UA (umol/l)	280 (114–374)	210 (117–332)	182 (123–315)	0.222
CRP (mg/l)	0.50 (0.37–1.46)	0.48 (0.38–1.90)	0.46 (0.38–1.01)	0.764
CTRP4 (ng/ml)	5.50 (4.38–9.42)	4.94 (3.01–9.20)	4.06 (2.84–5.76)	0.001
Medications (n, %)				
Insulin	29 (51.8%)	30 (55.6%)	31 (56.4%)	0.875
Oral antidiabetic drugs only	25 (44.6%)	22 (40.7%)	22 (40.0%)	0.868
Anti-platelet	18 (32.1%)	17 (31.5%)	12 (21.8%)	0.405
Statin	22 (39.3%)	15 (27.8%)	23 (41.8%)	0.268
Antihypertensive drugs	36 (64.3%)	28 (51.9%)	23 (41.8%)	0.059
*β*-blocker	8 (14.3%)	11 (20.4%)	11 (20.0%)	0.648
ACEI/ARB	26 (46.4%)	17 (31.5%)	14 (25.5%)	0.057
Calcium channel blocker	28 (50.0%)	16 (29.6%)	15 (27.3%)	0.023
diuretic	12 (21.4%)	7 (13.0%)	3 (5.5%)	0.040

Data were expressed as mean ± SD for normally distributed continuous variables, median (interquartile range) for abnormally distributed variables and number (%) for category variables. ANOVA or Kruskal–Wallis (KW) test was performed among groups for continuous variables and Chi-square test was used for categorical variables

*BMI* body mass index, *DM* diabetes mellitus, *SBP* systolic blood pressure, *DBP* diastolic blood pressure, *FBG* fasting blood glucose, *PBG* postprandial blood glucose, *HbA1c* hemoglobin A1c, *TC* total cholesterol, *TG* triglyceride, *HDL-C* high-density lipoprotein cholesterol, *LDL-C* low-density lipoprotein cholesterol, *UA* uric acid, *CRP* C reactive protein, *ACEI* Angiotensin converting enzyme inhibitor, *ARB* Angiotensin receptor blocker

### Increased CTRP4 level was an independent factor for low FMD in T2DM patients

According to Table 2, there was an independent correlation between total CTRP4 amount and low FMD in T2DM cases after adjusting for sex, age, BMI, smoking, drinking, hypertension, serum creatinine and UA (OR = 1.03 95% CI 1.01–1.05) (Model 1), as well as further adjustment for diabetes course, HbA1c, LDL-C and TG (OR = 1.03 95% CI 1.00–1.06) (Model 2) and intake of oral antidiabetic drugs and antihypertensive drugs (OR = 1.03, 95% CI 1.00–1.05) (Model 3). When compared to tertile 1, the odds ratios (ORs) for tertile 2 and tertile 3 of serum CTRP4 were 4.60 (95% CI 1.77–11.98) and 5.25 (95% CI 1.94–14.25) in Model 1, respectively. The corresponding ORs and 95% CIs were 4.87 (1.72–13.81) and 6.01 (2.05–17.63) in adjusted Model 2 and 4.78 (1.69–13.55) and 5.99 (2.04–17.65) in adjusted Model 3, respectively (all p for trend < 0.01). Every 1SD increase in CTRP4 (ng/ml) was associated with 1.89-fold (95% CI 1.11–3.23) increased risk of low FMD after adjusting for sex, age, BMI, smoking, drinking, hypertension, serum creatinine and UA (Model 1). After further adjustment for diabetes course, HbA1c, LDL-C and TG as well as the use of oral antidiabetic drugs and antihypertensive drugs, similar results were obtained (OR = 1.90, 95% CI 1.10–3.28, Model 2; OR = 1.88, 95% CI 1.08–3.26, Model 3).

**Table 2:** Association of CTRP4 levels with low FMD in diabetic patients

CTRP4	Model 1		Model 2		Model 3	
	OR	95%CI	OR	95%CI	OR	95%CI
Total	1.03	1.01–1.05	1.03	1.00–1.06	1.03	1.00–1.05
Tertiles						
1st	Ref	–	Ref	–	Ref	–
2nd	4.60	1.77–11.98	4.87	1.72–13.81	4.78	1.69–13.55
3rd	5.25	1.94–14.25	6.01	2.05–17.63	5.99	2.04–17.65
P trend	P < 0.01 P < 0.01 P < 0.01					
SD	1.89	1.11–3.23	1.90	1.10–3.28	1.88	1.08–3.26

Model 1: Adjusted for sex, age, BMI, smoking, drinking, hypertension, serum creatinine and UA

Model 2: Additional adjustment for diabetes course, HbA1c, LDL-C and TG in Model 1

Model 3: Additional adjustment for intake of oral antidiabetic and antihypertensive drugs in Model 2

Tertile 1 of CTRP4 levels was taken as a reference in the binary logistic regression analysis

### Predictive significance of serum CTRP4 for low FMD in T2DM cases

**Fig. 3:**
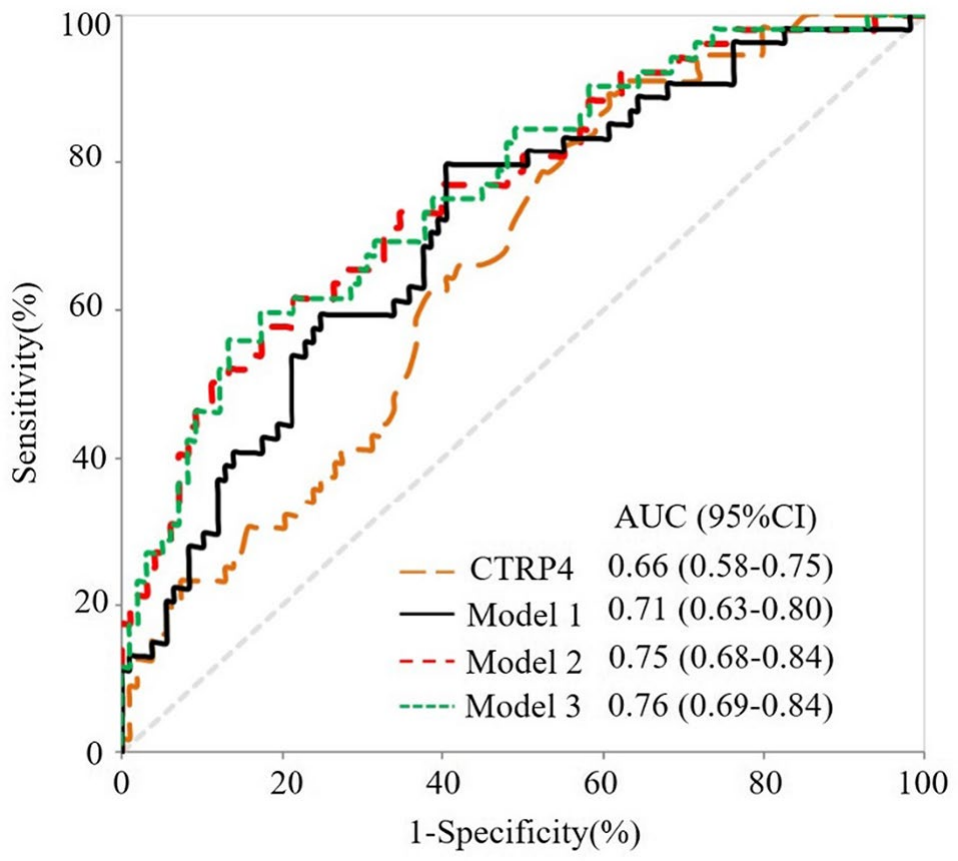
Receiver operating characteristic (ROC) curves for low FMD in T2DM patients. AUC, areas under the ROC curve; CI: confidence interval

The areas under the ROC curves (AUCs) for Model 1, Model 2 and Model 3 were 0.71, 0.75 and 0.76, respectively; there were no significant differences between the AUCs based on three different models and that based on CTRP4 (AUC = 0.66, 95% CI 0.58–0.75) (all *p* > 0.05). In addition, ROC analysis showed that the threshold of CTRP4 in predicting low FMD was 3.76 ng/ml, with a sensitivity 89.3% and a specificity 39.4% (Fig. 3).

### Biological functions of CTRP4 on HUVECs

**Fig. 4:**
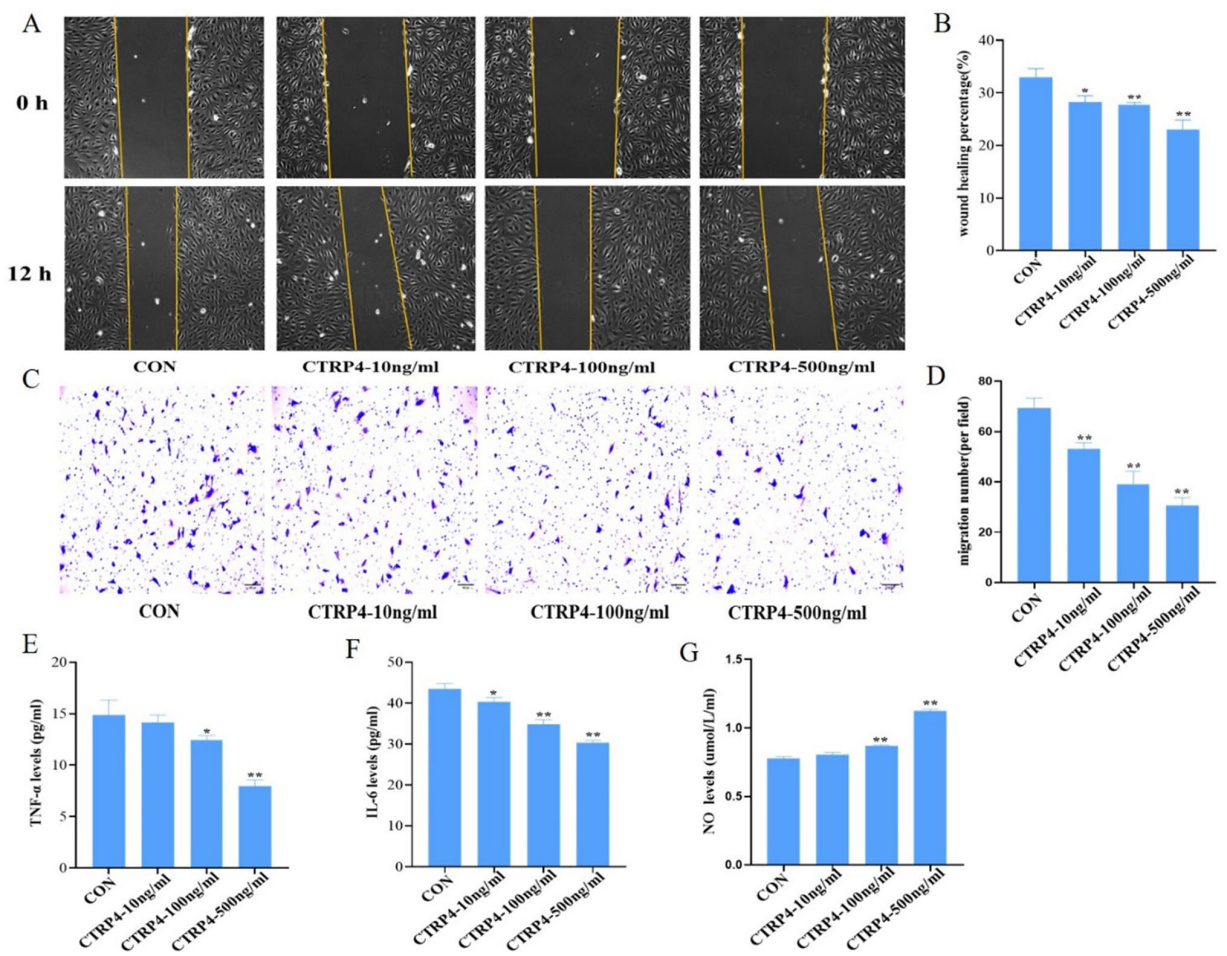
CTRP4 inhibits proliferation, migration and inflammation in HUVECs (*n* = 3). **A** Wound healing assay was performed in HUVECs upon stimulation with CTRP4 of increasing concentrations (10, 100 and 500 ng/ml). Images were taken before and 12 h after cell scratch. **B** Quantification of results in A was calculated as described in methods. **C** Transwell assay of HUVECs was performed after starvation. CTRP4 of increasing concentration (10, 100 and 500 ng/ml) were used to stimulate migration. The migrated cells were quantified by crystal violet staining. **D** Quantification of experiment results in C. **E**–**G** Levels of TNF-*α*, IL-6 and NO in HUVECs treated with CTRP4 of increasing concentrations (10, 100 and 500 ng/ml). * *p* < 0.05 versus CON group ** *p* < 0.01 versus CON group

**Fig. 5:**
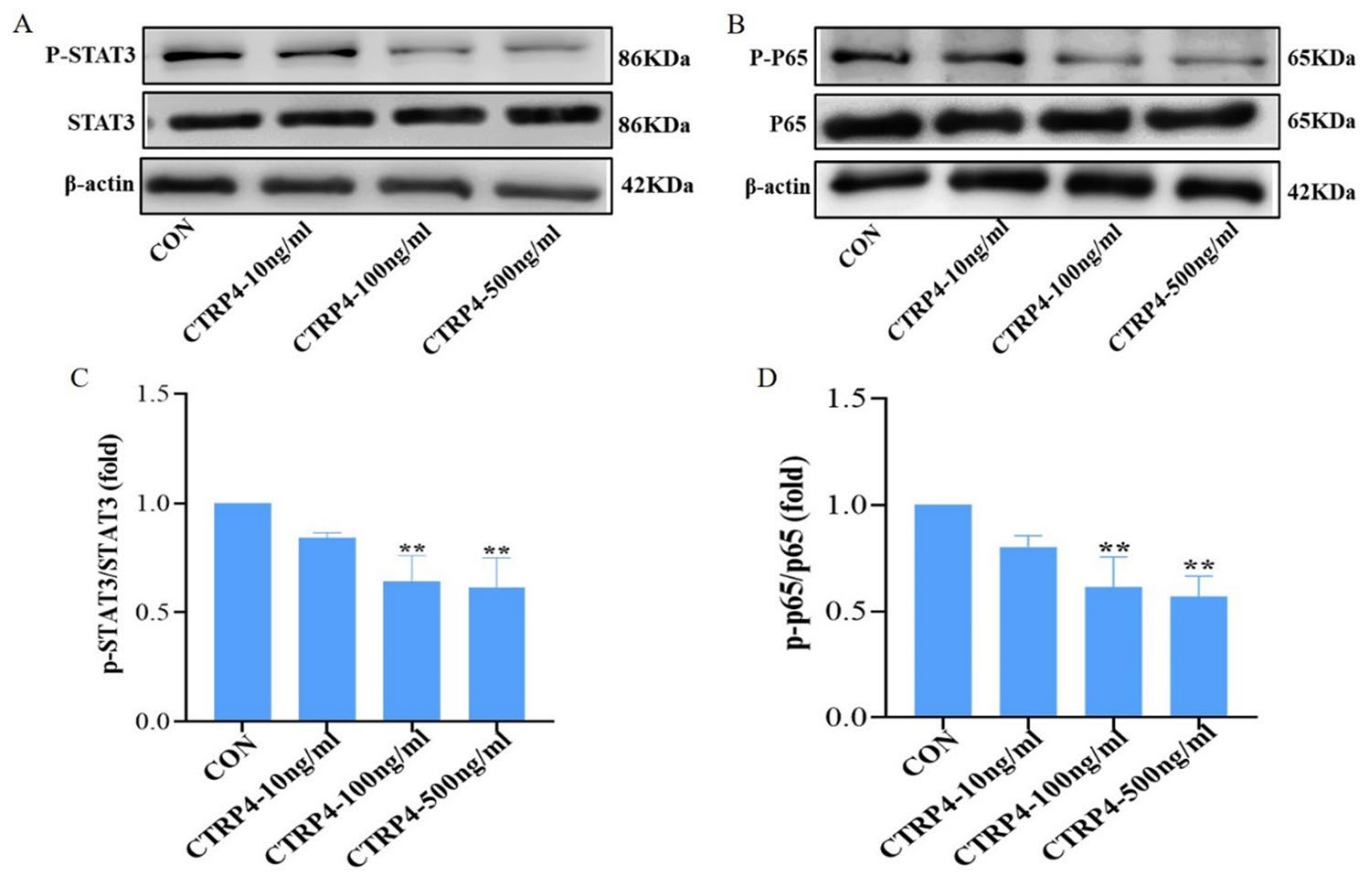
Effects of CTRP4 and pathways (*n* = 3). **A–B** HUVECs were treated with CTRP4 of increasing concentration (10, 100 and 500 ng/ml) for 24 h and Western blot was performed to examine the expression of related proteins. **C–D** Quantification of Western blot results in A and B, ** *p* < 0.01 versus CON group

We further performed function assay upon CTRP4 stimulation in HUVECs. In wound healing and mobility test, CTRP4 inhibited proliferation and migration of HUVECs in a concentration-dependent manner (all *p* < 0.05) (Fig. 4A–D). ELISA Kit showed that CTRP4 could significantly down-regulate the expression of IL-6, TNF-*α* and remarkably increase the levels of NO in the supernatant with the concentration of 100 ng/ml and 500 ng/ml (all *p* < 0.05) (Fig. 4E–G). Moreover, this protein suppressed pathways including NF-κB and STAT3 (downregulating expression of p-STAT3 and p-p65) (Fig. 5). These results indicated that CTRP4 could protect against proliferation, migration and inflammation in HUVECs.

## Discussion

Type 2 diabetes mellitus (T2DM), characterized by endothelial dysfunction and vascular remodeling, is a well-known risk factor for the development of cardiovascular diseases [32]. As a matter of fact, patients with T2DM have up to four times increased risk of developing cardiovascular events than patients without T2DM [33]. Abnormalities in the production of nitric oxide (NO) by the vascular endothelium have been linked to endothelial dysfunction, which is believed to play a crucial role in the initial phase of atherosclerosis. It worsens throughout the entire progression of atherosclerosis, which is notably exacerbated in individuals with diabetes [34]. Flow-mediated dilation (FMD) on peripheral brachial artery with high-resolution ultrasound is one of commonly employed methods for examining endothelial function in clinical. Meta-analysis concluded that every 1% increase in FMD was associated with an approximately 8–13% reduction in the odds of cardiovascular events [35]. Therefore, FMD monitoring may have an important prognostic significance for diabetic angiopathy.

Previous studies indicated that hyperlipidemia, hyperglycemia, cigarette smoking, elevated BMI, hypertension and metabolic syndrome were risk factors for endothelial dysfunction [36–40]. In our study, no significant differences in baseline characteristic indicators, such as BMI, smoking, hypertension, serum lipids and blood glucose were observed in three different FMD groups, which may be attributed to the small sample size in this research. It was reported by Pronko et al. [41] that 23.5–27% patients with II grade hypertension, as well as 75% of patients with III grade hypertension, had endothelial dysfunction. Similarly, we observed that the higher SBP was associated with a lower FMD value. A variety of antihypertensive agents have been reported to exert protective effects against endothelial dysfunction [42]. Miroslav et al. [43] discovered that calcium channel blocker was efficient for the enhancement of FMD parameters. Yousef et al. [44] reported that the use of angiotensin converting enzyme inhibitor/angiotensin receptor blocker (ACEI/ARB) for hypertension could effectively improve FMD in comparison to other antihypertensive agents. Diuretics have been reported to reduce peripheral arterial resistance in humans and promote endothelium dependent relaxations [45]. In the present study, the percentage of patients using calcium channel blocker or diuretics were greater in low FMD group than middle and high FMD group; however, the proportion of patients taking ACEI/ARB among three FMD groups did not achieve statistical significance.

Unlike other family members, CTRP4 contained two C1q globular domains which endowed it with diverse physiological functions. The research by Dai et al. [46] claimed that acute coronary syndrome (ACS) patients had higher serum CTRP4 levels than non-ACS cases. However, as compared with control subjects, serum CTRP4 levels were lower in patients with T2DM [47]. The current investigation found a negative correlation between serum CTRP4 concentrations and FMD in individuals with T2DM. Additionally, logistic regression analysis revealed that elevated CTRP4 levels independently contributed to reduced FMD in diabetic patients. Nevertheless, the specific mechanisms linking elevated CTRP4 levels and increased risk of low FMD remained largely unclear. Li Q et al. [48] found that CTRP4 could enhance the expression of interleukin-6 (IL-6) and stimulate the nuclear factor-κB (NF-κB) and signal transducer and activator of transcription 3 (STAT3) signaling pathways. Li Y et al. [25] reported that CTRP4 suppressed appetite through inducing the STAT3 and NF-κB pathways. Interestingly, Ye et al. [49] indicated that central Ad-CTRP4 intervention could decrease the level of tumor necrosis factor-*α* (TNF-*α*) and IL-6 in both hypothalamus and peripheral. In macrophages, CTRP4 has been reported to inhibit the activation of NF-κB/P65 and STAT3, as well as the production of inflammatory cytokines [27]. These controversial studies manifested that the roles of CTRP4 were contingent on the circumstances and could act both as an anti-inflammatory and as a pro-inflammatory protein during different stages of the disease. CTRP4 may exert pro-inflammatory effects in cancer-related inflammation, while act as an anti-inflammatory agent in other inflammatory environments [50]. These conflicting results may also be associated with the fact that CTRP4 could bind to different cellular receptors and thus play different physiological and pathological roles [50]. In our cellular experiments, CTRP4 could inhibit proliferation and migration of HUVECs in vitro. This protein was also able to down-regulate the expression of IL-6, TNF-*α* and increase the level of NO through suppressing STAT3 and NF-κB pathways in HUVECs. STAT3 and NF-KB have been implicated as major transcription factors in numerous cellular processes, involved in cell growth, inflammation and apoptosis [51]. These results demonstrated that endothelial dysfunction may contribute to increased serum CTRP4 levels and CTRP4 was critical to the pathophysiology of vascular function.

## Limitations of the study

A number of limitations of this study should be discussed when considering the results. First, a causal relationship between CTRP4 and FMD could not be concluded due to the cross-sectional study design. Second, since this was a single-center study and had relatively limited sample size, it was not fully representative of the general population. Third, the effects of nitroglycerin on endothelial independent vasodilatation were not assessed and vascular morphology was not evaluated. Finally, HUVECs may not be the perfect cellular model to investigate the influences of CTRP4 on vascular endothelial function and crosstalk between NF-κB and STAT3 pathways in our experiments were not analyzed. In the future, prospective study with regard to the links of endothelial function and CTRP4 level will be conducted. Moreover, additional in vivo studies are required to clarify the mechanisms mediating CTRP4 and endothelial function.

## Conclusion

In conclusion, we observed that T2DM patients with reduced FMD exhibited elevated levels of serum CTRP4. However, CTRP4 could alleviate proliferation, migration and inflammation by suppressing NF-κB and STAT3 signaling pathways in HUVECs. Our findings have suggested a notion that elevated serum CTRP4 levels may occur as the result of endothelial dysfunction, and CTRP4 could be applied as a useful marker for monitoring endothelial dysfunction.

## Data Availability

The original data used in this study are available from the corresponding author upon request.

## Abbreviations

CTRP4: C1q/TNF-related protein 4
FMD: Flow-mediated dilatation
T2DM: Type 2 diabetes mellitus
LPS: Lipopolysaccharides
HUVECs: Human umbilical vein endothelial cells
AGEs: Advanced glycation end products
NO: Nitric oxide
IL-6: Interleukin-6
TNF-α: Tumor necrosis factor-α
CTRPs: C1q/TNF-related proteins (CTRPs)
ROC: Receiver operating characteristic curve
AUC: Area under the ROC curve
OR: Odds ratio
SD: Standard deviation
CAD: Coronary artery disease
ACS: Acute coronary syndrome
BP: Blood pressure
SBP: Systolic blood pressure
DBP: Diastolic blood pressure
BMI: Body mass index
CRP: C reactive protein
HbA1c: Glycosylated hemoglobin A1c
FBG: Fasting blood glucose
PBG: Postprandial blood glucose
TC: Total cholesterol
HDL-C: High-density lipoprotein cholesterol
TG: Triglyceride
LDL-C: Low-density lipoprotein cholesterol
UA: Uric acid
ACEI: Angiotensin converting enzyme inhibitor
ARB: Angiotensin receptor blocker
NF-κB: Nuclear factor-κB
STAT3: Signal transducer and activator of transcription 3
